# Increased Breast and Colorectal Cancer Risk in Type 2 Diabetes: Awareness Among Adults With and Without Diabetes and Information Provision on Diabetes Websites

**DOI:** 10.1093/abm/kaac068

**Published:** 2023-03-09

**Authors:** Laura Ashley, Kathryn A Robb, Daryl B O’Connor, Rebecca Platt, Mollie Price, Olivia Robinson, Elizabeth Travis, Lorraine Lipscombe, Ramzi Ajjan, Rebecca Birch

**Affiliations:** School of Humanities & Social Sciences, Leeds Beckett University, UK; Institute of Health and Wellbeing, University of Glasgow, UK; School of Psychology, University of Leeds, UK; School of Humanities & Social Sciences, Leeds Beckett University, UK; School of Humanities & Social Sciences, Leeds Beckett University, UK; School of Humanities & Social Sciences, Leeds Beckett University, UK; School of Psychology, University of Leeds, UK; Department of Medicine, University of Toronto, Canada; School of Medicine, University of Leeds, UK; School of Medicine, University of Leeds, UK

**Keywords:** Diabetes, Cancer, Awareness, Risk perception, Health information, Cancer screening

## Abstract

**Background:**

People with type 2 diabetes mellitus (T2DM) have a higher risk of developing breast and bowel cancers but are less likely to participate in cancer screening.

**Purpose:**

Two interlinked studies examined public awareness of the fact that T2DM increases breast and bowel cancer risk, and provision of this information on diabetes websites.

**Methods:**

Study-1: phase-1 surveyed awareness of T2DM-increased cancer risk in a nationally-representative British sample aged 50–74 (*N* = 1,458) and compared respondents with and without T2DM (*n* = 125 vs. *n* = 1,305); phase-2 surveyed an additional exclusively T2DM sample (*N* = 319). Study-2: High-ranking diabetes websites (*N* = 25) were reviewed to determine the rate of inclusion of cancer risk and cancer screening information in evident sections about diabetes-related health conditions.

**Results:**

A low proportion of respondents were aware that T2DM increases risk of breast (13.7%) and bowel (27.6%) cancers, compared to much higher awareness of other diabetes-related conditions such as sight loss (82.2%) and foot problems (81.8%). Respondents with T2DM were significantly more likely than those without T2DM to be aware of all the surveyed diabetes-related health conditions (e.g., sight loss, OR: 3.14, 95%CI: 1.61–6.15; foot problems, OR: 2.58, 95%CI: 1.38–4.81), except breast (OR: 0.82, 95%CI: 0.46–1.45) and bowel (OR: 0.95, 95%CI: 0.63–1.45) cancer, for which awareness was equally low among people with and without T2DM. Few diabetes websites with a section on diabetes-related health conditions included cancer in this section (*n* = 4/19), and fewer still included cancer screening among any noted cancer-protective behaviors (*n* = 2/4).

**Conclusions:**

There is low public awareness that T2DM increases the risk of developing breast and bowel cancers, even among people with T2DM, which may be partly due to limited information provision regarding T2DM-increased cancer risk from diabetes care providers and organizations.

## Teaser

Most people are not aware that Type 2 diabetes increases the risk of breast and bowel cancers. Awareness is low even among people who have Type 2 diabetes. This may be partly because little information about cancer risk is being provided by diabetes care organisations.

The incidence and prevalence of diabetes mellitus (DM) are high and increasing worldwide, with type 2 DM (T2DM) accounting for over 90% of all DM cases [1]. In the United Kingdom, 3.4 million people have T2DM, which is approximately 10% of people aged over 40, with a further 1 million yet to be diagnosed; these combined figures are projected to increase to 4.9 million by 2030 [2]. T2DM is associated with advancing age and is a risk factor for several microvascular and macrovascular health complications, such as heart disease, foot problems that can lead to amputation, and retinopathy which can cause sight loss [1, 3]. People with diabetes are approximately twice as likely to develop heart disease, and 20 times more likely to experience an amputation, and diabetes is a leading cause of preventable sight loss [3–5]. People with T2DM also have an increased lifetime risk of numerous cancers, including liver, pancreatic, endometrial, bladder, and kidney cancers, as well as screening-amenable breast and colorectal cancers, independent of confounding factors such as obesity [6, 7]. Recent reviews show diabetes-increased risks of around 20% for breast cancer [6–8] and 30% for colorectal cancer (CRC) [6–8]. Furthermore, cancer patients with diabetes are more likely to experience complications and side-effects during cancer treatments and have poorer survival [9–11]. In recent years high-income countries have seen decreasing rates of vascular complications (e.g., myocardial infarction, stroke, and amputation), which has led to diversification in diabetes complications and causes of death, such that in some countries or regions cancer is now the most common cause of death among people with diabetes [3, 12, 13].

This article presents two complementary studies concerning the increased risk of cancer among people with T2DM. In the first study, we examine public awareness of the fact that T2DM increases the risk of breast and CRC, relative to awareness of other diabetes-related health conditions, such as heart disease and sight loss. Diabetes care providers and organizations are a key source of information for patients and the public about diabetes-related health risks. Therefore, in the second study, we examined provision of the information that T2DM increases cancer risk from diabetes care providers and organizations, relative to information provision about other diabetes-related health conditions such as heart disease and sight loss.

Breast mammography and fecal-based CRC screening tests aim to detect presymptomatic early-stage cancer, which is associated with superior treatment and survival outcomes [14, 15]. In the United Kingdom, for example, 5-year net survival for breast cancer diagnosed at stages 1, 2, 3, and 4 is 98%, 90%, 72%, and 26% respectively [16]; equivalent figures for CRC are 92%, 84%, 65%, and 10% [17]. Despite their increased cancer risk, people with T2DM are less likely to participate in organized nationally offered cancer screening programs [18–21] like those in the UK [22]. For example, von Wagner et al. [20] found that adults with T2DM in England were significantly less likely to have ever completed (63% vs. 76%), or to be up-to-date with (60% vs. 72%), biennial fecal-based CRC screening compared to people without T2DM, even after controlling for confounding factors (e.g., socioeconomic status (SES)). For people with T2DM, lack of awareness that T2DM increases breast and CRC risk precludes fully informed decision-making about cancer screening participation and, as risk perception is a theoretical and empirical driver of volitional health behaviors [23–26], may be a contributory factor to non-uptake. A recent review of barriers to CRC screening among individuals at higher risk due to family history concluded lack of awareness of being at higher risk of CRC is a main barrier to undergoing screening [27]. For people with T2DM, cancer risk awareness is also important to informing decisions and motivations around health behavior changes aimed at achieving diabetes remission (e.g., healthier diet and more exercise); this is similarly the case for people with prediabetes, at high risk of developing T2DM, in relation to behavioral efforts to prevent diabetes.

Research shows low public awareness of several cancer risk factors including alcohol and obesity [28–30]. To our knowledge, no research has examined public awareness of T2DM-increased risk of breast cancer, and limited research, solely using the Bowel (/Colorectal) Cancer Awareness Measure (CAM) [31], has examined lay knowledge of T2DM-increased risk of CRC. Studies using the Bowel-CAM have consistently found “having diabetes” to be the least well-recognized of the measure’s CRC risk factors (e.g., [31–33]), though none of these studies appear to have examined T2DM-status as a correlate of awareness nor recruited an exclusively T2DM-sample. Therefore, study 1 aimed, for the first time, to examine awareness of T2DM-increased breast and CRC risk among British adults with and without T2DM.

A key component of diabetes education is raising awareness of diabetes health complications (e.g., diabetic retinopathy) and promoting the uptake of associated protective health behaviors (e.g., retinopathy screening). As recent years have seen “large reductions in classic complications of T2DM in high-income countries” [p. 537 [34],], there have been calls for care providers to adopt a more “holistic view” of diabetes complications which includes “often-overlooked” health problems such as the increased risk of cancer [8, 35]. Indeed, given that some countries have seen “a transition from vascular diseases to cancers as the leading contributor to diabetes-related death” [pp. 165, 12], there have been calls for the promotion and even inclusion of cancer screening within the context of diabetes education and care [36, 37]. Thus, as a complement to examining levels of public awareness of T2DM-increased cancer risk, we sought to examine to what extent diabetes care providers are including cancer within their information provision on diabetes-related health risks. We focused on online information as most diabetes care providers and organizations now provide much or all their information via a website. Internet-based information is a major source of health knowledge for the public [38–40], and a systematic review of diabetes-related information-seeking behavior found the Internet was the most frequently reported information source [38]. Also, clinicians increasingly consult online information and signpost patients and carers to website-based resources [41–43]. Few studies have analyzed diabetes websites, and none appear to have examined the provision of information about cancer risk or cancer screening [44–46]. Study 2 aimed to address this gap by examining, among a sample of high-ranking diabetes websites, how many include breast and CRC in information about diabetes-related health conditions and, of those that do, how many include cancer screening among any listed protective health behaviors. Understanding to what extent key public information on diabetes-related health risks includes cancer (study 2) may help explain, and identify a potential target for raising, public awareness of T2DM-increased cancer risk (study 1) (i.e., knowledge level may reflect the level of information provision).

This research aimed to examine public awareness of the fact that T2DM increases breast and CRC risk, and the provision of this information from diabetes care providers and organizations via their websites. To address these aims, we conducted two interlinked novel studies, and present here their complementary findings. In the first study, phase-1, we surveyed awareness of T2DM-increased cancer risk in a nationally representative sample of British adults aged 50–74 (corresponds with the eligible-age for breast and CRC screening in Britain), and compared awareness between those with and without T2DM. As adults with T2DM are a relatively small subgroup of the general population, to replicate the phase-1 survey findings among the T2DM-subgroup in a larger sample, in phase-2 we surveyed an additional exclusively T2DM sample. To help explain and potentially address the findings of the first study (concerning public awareness of T2DM-increased cancer risk), in the second study we reviewed the rate of inclusion of cancer risk and cancer screening information, in evident sections about diabetes-related health conditions, on diabetes websites high-ranking in British-based internet searches.

## Methods

### Study 1: Public Awareness Survey

#### Phase-1: Nationally representative general population sample

##### Survey design and conduct

In July–August 2020 we added questions to a computer-assisted telephone omnibus survey, run by market research company Ipsos MORI, which is carried out weekly by trained interviewers with a different nationally representative sample of British adults.

##### Participants, sampling, and recruitment

Adults aged 50–74 living in Britain were eligible to participate, provided they were able to speak English and give informed consent. Proportional quota sampling was used with quotas on sociodemographic characteristics (e.g., gender, age, ethnicity, region; see footnotes to Table 1) according to the known profile of Britain based on data from the Publishers Audience Measurement Company (data wave April 2019—March 2020). Participants were recruited by random digit dialing of landline and mobile telephone numbers 9 am–9 pm weekdays and 10 am–7 pm weekends; interviewers did not leave messages with non-responding telephone numbers.

**Table 1:** Summary of the Characteristics of the Survey Samples in Study 1, *n* (%)

	Phase-1			Phase-2
	Nationally representative^†^ total sample (*N* = 1,458)	The total sample weighted	Subgroup with T2DM (*n*= 1 25)	Exclusively T2DM sample (*N*= 319)
**Gender**				
Female	749 (51.4)	744 (51.1)	57 (45.6)	125 (39.2)
Male	707 (48.5)	712 (48.8)	68 (54.4)	193 (60.5)
decline to answer	2 (0.1)	2 (0.1)	0 (0)	1 (0.3)
**Age (years)**				
Mean ± SD	62.26 ± 7.24	61.55 ± 7.17	64.98 ± 6.80	63.43 ± 7.23
50-59	530 (36.4)	587 (40.3)	27 (21.6)	100 (31.3)
60-69	585 (40.1)	569 (39.0)	57 (45.6)	130 (40.8)
70-74	317 (21.7)	274 (18.8)	40 (32.0)	84 (26.3)
decline to answer^1^	26 (1.8)	28 (1.9)	1 (0.8)	5 (1.6)
**Socioeconomic status: social grade** ^2^				
ABC1	966 (66.3)	786 (53.9)	69 (55.2)	163 (51.1)
C2DE	448 (30.7)	639 (43.8)	55 (44.0)	151 (47.3)
Missing	44 (3.0)	33 (2.3)	1 (0.8)	5 (1.6)
**Ethnicity**				
White British or White other	1351 (92.7)	1338 (91.8)	116 (92.8)	296 (92.8)
Black or mixed Black or Black other	44 (3.02)	46 (3.2)	3 (2.4)	8 (2.5)
Asian or mixed Asian or Asian other	29 (2.0)	34 (2.3)	4 (3.2)	12 (3.8)
Other ethnicities	22 (1.5)	26 (1.8)	0 (0)	2 (0.6)
do not know	3 (0.2)	3 (0.2)	1 (0.8)	0 (0)
decline to answer	9 (0.6)	11 (0.7)	1 (0.8)	1 (0.3)
**Country of residence**				
England	1226 (84.1)	1247 (85.5)	95 (76.0)	257 (80.6)
Scotland	174 (11.9)	133 (9.1)	20 (16.0)	38 (11.9)
Wales	58 (4.0)	78 (5.3)	10 (8.0)	24 (7.5)
**Self-reported type 2 diabetes**				
Yes	125 (8.6)	126 (8.6)	125 (100)	319 (100)
No	1305 (89.5)	1306 (89.6)	–	–
do not know	7 (0.5)	6 (0.4)	–	–
decline to answer	21 (1.4)	20 (1.4)	–	–

^1^People who declined to give their precise age, though confirmed they were aged 50–74.

^2^Social grades “ABC1” include professional, managerial, and non-manual occupations, and “C2DE” skilled manual and unskilled occupations and the long-term unemployed.

^†^Sampling quotas were based on data from the Publishers Audience Measurement Company (data wave April 2019—March 2020), and were as follows: gender: female (51.1%), male (48.9%); age: 50–54 (23.8%), 55–64 (41.9%), 65–74 (34.3%); socioeconomic status: AB (27.4%), C1 (28.8%), C2 (20.5%), D (13.05%), E (10.2%); ethnicity: white (91.8%), ethnic minority (8.2%); country of residence: England (85.5%, with 9 regional sub-quotas), Scotland (9.2%), Wales (5.3%), and additionally, not shown in Table 1, working status: working (50.4%), not working (49.6%). These are proportions for the whole sample; gender-based sub-quotas were further set for each variable.

##### Survey questions

Awareness of T2DM-increased cancer risk, relative to other diabetes-related health problems, was assessed with open and closed questions typical of those used in previous research [e.g., 29, 47]. Respondents were first asked the open question “*are there any medical conditions or illnesses that people with type 2 diabetes are more likely to develop at some point in the future – compared to people who don’t have type 2 diabetes?*” Respondents were asked to “*name as many as you can think of*” and prompted with “*anything else”*. Interviewers then said “*it can be difficult to think of medical conditions and illnesses off the top of your head, so we have a list of these for you to consider*”. Respondents were asked if people with T2DM are more likely or not, compared to those without T2DM, to develop each of nine health conditions: sight loss, foot problems, heart disease, nerve damage, dementia, breast cancer and bowel cancer, and two “decoys” cervical cancer and prostate cancer (for which T2DM does not increase risk [6, 7, 48]). The health conditions were chosen based on research evidence and those listed on the Diabetes UK website [3, 48], and were listed in a different computer-randomized order for each respondent. Response options were “*more likely*”, “*not more likely*”, “*don’t know*”, and decline to answer. Respondents were also asked about their sociodemographic characteristics, and if they have T2DM (see Table 1).

##### Ethics

The survey received ethical approval from the Leeds Beckett University Psychology Local Research Ethics Committee (ref:72272). Ipsos MORI operate in accordance with the international quality standard for market research (ISO 20252) and UK Data Protection Act (2018).

##### Analysis

Data were analyzed using IBM-SPSS-Statistics version-26 and Stata version-15.0. Descriptive analysis was undertaken to examine sample characteristics, and awareness of T2DM-increased breast and CRC risk comparative to awareness of other diabetes-related health conditions. To assess correlates of awareness, separate multivariable logistic regression models, including diabetes status, gender, age, SES (using occupation-based social grade, which is strongly associated with education level [49, 50]), and ethnicity (entered in a single step, see Table 3 for how these variables were categorized), were produced for each of the seven diabetes-related health conditions examined in the closed question (plus decoys) to estimate odds-ratios (OR) and 95% confidence intervals (CI). Binary outcome variables (aware/not aware) were derived from the closed question response options with “*more likely*” responses coded aware and “*not more likely*” and “*don’t know*” responses coded not aware (except for the decoy items, where the “*more likely*” and “*not more likely*” responses were swapped round; see footnote to Table 3). Thus we compared “know” vs. “don’t know” (where “don’t know” is the combination of the “*don’t know*” response option plus do not know as indicated by the incorrect response option), which we consider a more interpretable and meaningful comparison than “*don’t know*” response option vs. a combination of the “*more likely*” plus “*not more likely*” responses (i.e., correct and incorrect responses combined). Decline to answer was coded as missing data. All analyses were performed with and without sample weights provided by Ipsos MORI to correct for small deviations from sampling quotas representative of the British population; as findings were not substantively different with inclusion of the weights, we present the results of the unweighted analyses.

#### Phase-2: Exclusively-T2DM sample

In phase-2, January–February 2021, only survey respondents who reported that they have T2DM (in response to the diabetes status question) were asked the awareness questions about diabetes-related health problems. The methods and analysis were identical to phase-1 except, as all respondents had T2DM, diabetes status was not examined as a correlate of awareness.

### Study 2: Diabetes Websites Review

#### Selecting a sample of websites

We searched for diabetes websites using the top three search engines with the greatest UK and global market share (Google, Bing, and Yahoo) [51]. For each of eight search terms (diabetes, diabetes mellitus, type 2 diabetes, diabetes signs, diabetes symptoms, diabetes treatment, diabetes websites, diabetes education) the first 25 results were examined (paid advertisements were not treated as search results). This search strategy resembles how people typically search for health-related information online (via search engines, using one or two keywords, and viewing only the initial results) [52, 53], and is consistent with sampling strategies employed in previous research aiming to identify websites likely to be accessed by the public [46, 54]. Search results were excluded if: (1) non-English language; (2) not a website (e.g., YouTube video); (3) website inaccessible (e.g., requires a login, deactivated); (4) not a diabetes-specific website (e.g., bhf.org.uk); (5) diabetes-specific website but concerned *exclusively* with: (5a) non-T2DM, (5b) diabetes in children and young people, (5c) one specific aspect of diabetes such as healthy eating, (5d) personal experiences (e.g., blogs), (5e) resources and/or training for professionals (e.g., cdep.org.uk); and (6) duplication (e.g., eligible website, but already identified).

Three researchers (RP, OR, and ET) independently searched for eligible websites in May 2020, each using a different one of the three search engines. Each researcher compiled a list of websites for inclusion and a list of any websites for which they were unsure of eligibility; a fourth researcher (LA) collated these lists and made decisions on any eligibility queries, if necessary via discussion with the other researchers until a consensus was reached. All websites identified by one or more of the researchers and judged eligible were included. Top-ranking search-engine results for diabetes websites include health-related websites which are not diabetes-specific, but have a diabetes section (e.g., nhs.uk). Therefore, we also selected for review the first three search results from each of the three search engines that were health-related but not diabetes-specific websites, from a search undertaken by LA using the term “diabetes” (i.e., up to nine websites in the unlikely event none of the results were duplicates). For all searches, we used Google Chrome web-browser with an “incognito” window (to avoid user account information and browsing history influencing search results), and where required by the search engine, selected UK as the region setting.

#### Reviewing the websites

The sampled websites were reviewed June 2020 to March 2021 using a two-stage strategy. For each website, stage-one review was independently undertaken by four researchers (RP, MP, OR, and ET), and stage-two review by two researchers (RP and OR). In addition, in June 2022, both review stages were reconducted and the results updated, though the updates were limited to a handful of changes which did not materially alter the findings (MP, any updates agreed with LA). Stage-one, websites were browsed using the site map and/or navigation tabs to determine (yes/no): (1) does the site have an evident section about diabetes-related health conditions; if yes, (2) does it list each of the health conditions in Table 2 (excluding decoys); if breast or CRC are listed, (3) does it also list any cancer-protective behaviors; and if yes, (4) do these include cancer screening. We created archived URLs for the webpages reviewed in this principal analysis, using the *Wayback Machine*, in June 2022 when we updated the website review (see electronic Supplementary Material 1). Stage-two, websites were searched using the search function to determine if any cancer risk or cancer screening information was contained elsewhere on the site. Twenty-eight key words or phrases (e.g., cancer; risk of cancer; cancer screening; mammogram; poo) were searched one at a time, and up to the first 10 returned results were reviewed; if there was an option to order the search results, relevance (not date) was selected. For the health-related websites which were not diabetes-specific, only stage-one review was undertaken, focused on just the evidently diabetes-specific webpage(s).

**Table 2:** Descriptive Summary of Responses to the closed Survey Questions in Study 1, *n* (%)

		Phase-1			Phase-2
			Type 2 diabetes^1^		
Diabetes-related health conditions ordered highest to lowest awareness (in phase-1, excluding decoy items)		Total sample (*N* = 1,458)	No (*n* = 1,305)	Yes, T2DM subgroup (*n* = 125)	Exclusively T2DM sample (*N* = 319)
**Sight loss**	More likely*	1198 (82.2)	1069 (81.9)	115 (92.0)	288 (90.3)
	*Not more Likely*	48 (3.3)	42 (3.2)	5 (4.0)	13 (4.1)
	*Do not know*	199 (13.6)	188 (14.4)	5 (4.0)	18 (5.6)
	*Decline to answer*	13 (0.9)	6 (0.5)	0 (0)	0 (0)
**Foot problems**	More likely*	1193 (81.8)	1066 (81.7)	113 (90.4)	297 (93.1)
	*Not more likely*	48 (3.3)	42 (3.2)	5 (4.0)	11 (3.4)
	*Do not know*	203 (13.9)	190 (14.6)	7 (5.6)	11 (3.4)
	*Decline to answer*	14 (1.0)	7 (0.5)	0 (0)	0 (0)
**Heart disease**	More likely*	1133 (77.7)	1012 (77.5)	108 (86.4)	227 (71.2)
	*Not more likely*	62 (4.3)	54 (4.1)	7 (5.6)	37 (11.6)
	*Do not know*	250 (17.1)	233 (17.9)	10 (8.0)	55 (17.2)
	*Decline to answer*	13 (0.9)	6 (0.5)	0 (0)	0 (0)
**Nerve damage**	More likely*	872 (59.8)	764 (58.5)	97 (77.6)	232 (72.7)
	*Not more likely*	126 (8.5)	117 (9.0)	8 (6.4)	28 (8.8)
	*Do not know*	445(30.5)	415 (31.8)	20 (16.0)	59 (18.5)
	*Decline to answer*	15 (1.0)	9 (0.7)	0 (0)	0 (0)
**Dementia**	More likely*	455 (31.2)	397 (30.4)	53 (42.4)	76 (23.8)
	*Not more likely*	316 (21.7)	287 (22.0)	26 (20.8)	108 (33.9)
	*Do not know*	672 (46.1)	613 (47.0)	46 (36.8)	134 (42.0)
	*decline to answer*	15 (1.0)	8 (0.6)	0 (0)	1 (0.3)
**Bowel cancer**	More likely*	402 (27.6)	364 (27.9)	33 (26.4)	62 (19.4)
	*Not more likely*	355 (24.3)	315 (24.1)	37 (29.6)	115 (36.1)
	*Do not know*	688 (47.2)	619 (47.4)	55 (44.0)	142 (44.5)
	*Decline to answer*	13 (0.9)	7 (0.5)	0 (0)	0 (0)
**Breast cancer**	More likely*	200 (13.7)	183 (14.0)	15 (12.0)	23 (7.2)
	*Not more likely*	476 (32.6)	424 (32.5)	46 (36.8)	139 (43.6)
	*Do not know*	768 (52.7)	690 (52.9)	64 (51.2)	156 (48.9)
	*Decline to answer*	14 (1.0)	8 (0.6)	0 (0)	1 (0.3)
***Decoy items***					
**Cervical cancer**	*More likely*	133 (9.1)	114 (8.7)	16 (12.8)	23 (7.2)
	Not more likely*	498 (34.2)	443 (33.9)	50 (40.0)	132 (41.4)
	*Do not know*	809 (55.5)	738 (56.6)	58 (46.4)	164 (51.4)
	*Decline to answer*	18 (1.2)	10 (0.8)	1 (0.8)	0 (0)
**Prostate cancer**	*More likely*	246 (16.9)	217 (16.6)	24 (19.2)	41 (12.9)
	Not more likely*	433 (29.7)	389 (29.8)	38 (30.4)	123 (38.6)
	*Do not know*	765 (52.5)	692 (53.0)	63 (50.4)	155 (48.6)
	*Decline to answer*	14 (1.0)	7 (0.5)	0 (0)	0 (0)

^1^ *n* < 1,458 for diabetes status, as *n* = 6 responded *do not know* and *n* = 20 *declined to answer*, *correct answer.

#### Analysis

The website reviews were collated by LA, who examined and resolved any discrepancies by also reviewing the website in question and, if necessary, via discussion with the other researchers until a consensus was reached. Frequencies were used to describe the proportion of sampled websites listing cancer, comparative to other conditions, in an evident site section about diabetes-related health conditions, and listing cancer screening among any noted cancer-protective health behaviors. We also noted the proportion of websites containing information about cancer risk or screening elsewhere on the site outside of the evident section about diabetes-related conditions.

## Results

### Study 1: Public Awareness Survey

#### Phase-1: Nationally representative general population sample

##### Participants

*N* = 1,458 people were surveyed; sample characteristics are summarized in Table 1.

##### Awareness that T2DM is a risk factor for breast and colorectal cancer

To the open survey question, just 4.3% of respondents replied that people with T2DM have an increased risk of cancer, and no respondent specified *breast* or *bowel* cancer. In contrast, 30.0% of respondents correctly recalled that people with T2DM have an increased risk of heart problems, and 24.9% eye problems including sight loss, which were the two most frequent replies to the open question after *don’t know* (32.4%). As Table 2 shows, for the closed survey questions, breast cancer and bowel cancer were the two health conditions fewest respondents (13.7% and 27.6% respectively) correctly recognized as being *more likely* among people with T2DM than those without. Comparatively, over 80% of respondents were aware that T2DM confers increased risk of sight loss and foot problems, and many respondents also were aware of the increased risks of heart disease (77.7%) and nerve damage (59.8%). Awareness of T2DM-increased risk of dementia (31.2%) was similarly low to that for cancer. Though many respondents indicated that they *don’t know* if people with T2DM have increased risk or not of breast and bowel cancer, it is notable that these cancers were the two health conditions the greatest number of respondents incorrectly said were *not more likely* in people with T2DM. For the decoy items cervical and prostate cancer, 34.2% and 29.7% of respondents, respectively, correctly answered that these cancers are *not more likely* among people with T2DM. It is notable more respondents wrongly believed T2DM increases prostate cancer risk (16.9%) than correctly knew T2DM does confer increased risk of breast cancer (13.7%).

##### Diabetes status and sociodemographic correlates of awareness

As Table 3 shows, after adjustment for sociodemographic characteristics, respondents with T2DM were significantly more likely than those without T2DM to be aware that T2DM confers increased risk of sight loss (OR 3.14, 95%CI 1.61–6.15), foot problems (OR 2.58, 95%CI 1.38–4.81), heart disease (OR 2.11, 95%CI 1.23–3.61), nerve damage (OR 2.72, 95%CI 1.75–4.24), and dementia (OR 1.63, 95%CI 1.11–2.38). In contrast, awareness of T2DM-increased risk of breast (OR 0.82, 95%CI 0.46–1.45) and bowel (OR 0.95, 95%CI 0.63–1.45) cancers was not significantly higher among respondents with T2DM compared to those without T2DM. Correct responses to the decoy cancer items were also not significantly associated with diabetes status (cervical cancer: OR 0.71, 95%CI 0.40–1.25; prostate cancer: OR 0.91, 95%CI 0.56–1.47). As Table 3 shows, female gender, younger age (50–59 vs. 70–74), and higher SES were significantly associated with greater awareness of some of the diabetes-related health conditions. Similarly, female gender and higher SES were significantly associated with correct answers to the decoy items. An exception is that female gender was associated with lower awareness of T2DM-increased risk of breast and bowel cancer.

**Table 3:** Summary of Multivariate Logistic Regression Analyses to Examine Correlates of Awareness of Diabetes-Related Health Conditions in Study 1

		Sight loss OR (95% CI)	Foot problems OR (95% CI)	Heart disease OR (95% CI)	Nerve damage OR (95% CI)	Dementia OR (95% CI)	Bowel cancer OR (95% CI)	Breast cancer OR (95% CI)	Decoy item Cervical cancer^†^ OR (95% CI)	Decoy item Prostate cancer^†^ OR (95% CI)
**Phase-1**										
**Diabetes**	No	*Reference category*								
	Yes	3.14 (1.61–6.15) **	2.58 (1.38–4.81) **	2.11 (1.23–3.61)**	2.72 (1.75–4.24)**	1.63 (1.11–2.38)**	0.95 (0.63–1.45)	0.82 (0.46–1.45)	0.71 (0.40–1.25)	0.91 (0.56–1.47)
	Unknown^1^	0.22 (0.10–0.50)**	0.21 (0.10–0.48)**	0.25 (0.11–0.54)**	0.50 (0.23–1.09)	0.50 (0.19–1.34)	0.54 (0.20–1.46)	0.41 (0.10–1.79)	0.71 (0.20–2.55)	0.68 (0.24–1.90)
**Gender**	Male	*Reference category*								
	Female	2.13 (1.60–2.83)**	2.66 (2.00–3.55)**	1.07 (0.83–1.38)	1.55 (1.25–1.93)**	0.81 (0.64–1.01)	0.76 (0.61–0.96)*	0.70 (0.52–0.95)*	1.85 (1.28–2.69)**	1.62 (1.22–2.14)**
	Unknown^1^	^	^	^	1.25 (0.07–21.55)	^	2.86 (0.16–52.42)	^	^	^
**Age**	50–59	*Reference category*								
	60–69	0.78 (0.56–1.09)	0.82 (0.59–1.14)	1.00 (0.74–1.35)	0.96 (0.75–1.22)	1.15 (0.89–1.49)	0.82 (0.63–1.07)	0.89 (0.63–1.26)	1.12 (0.73–1.71)	1.22 (0.88–1.68)
	70–74	0.53 (0.36–0.77)**	0.61 (0.42–0.89)**	0.61 (0.44–0.86)**	0.74 (0.55–0.99)*	0.96 (0.70–1.31)	0.72 (0.52–1.00)*	0.83 (0.54–1.26)	0.99 (0.60–1.63)	1.02 (0.70–1.48)
	Unknown^1^	0.94 (0.32–2.72)	0.42 (0.16–1.06)	0.56 (0.23–1.37)	0.73 (0.32–1.66)	0.60 (0.22–1.64)	0.42 (0.14–1.28)	1.36 (0.48–3.82)	0.60 (0.18–1.93)	1.05 (0.34–3.22)
**SES**	ABC1	*Reference category*								
	C2DE	0.50 (0.38–0.67)**	0.54 (0.41–0.73)**	0.70 (0.54–0.92)**	0.79 (0.63–1.00)*	1.12 (0.88–1.42)	1.01 (0.79–1.30)	1.28 (0.93–1.76)	0.52 (0.35–0.75)**	0.66 (0.49–0.88)*
	Unknown^1^	0.45 (0.22–0.91)*	0.53 (0.26–1.10)	0.76 (0.37–1.53)	0.62 (0.33–1.17)	1.03 (0.51–2.07)	1.03 (0.50–2.12)	1.41 (0.60–3.32)	0.39 (0.16–0.94)*	0.85 (0.36–2.00)
**Ethnicity**	White	*Reference category*								
	Non-White	0.69 (0.40–1.18)	1.52 (0.80–2.88)	1.06 (0.62–1.79)	1.09 (0.70–1.70)	0.96 (0.60–1.52)	0.73 (0.44–1.21)	1.06 (0.58–1.92)	0.96 (0.46–2.00)	1.50 (0.80–2.83)
	Unknown^1^	0.82 (0.20–3.33)	1.96 (0.39–9.91)	0.22 (0.07–0.73)**	0.55 (0.16–1.83)	0.74 (0.19–2.81)	0.23 (0.03–1.78)	1.01 (0.21–4.83)	0.78 (0.15–3.95)	1.37 (0.29–6.47)
**Phase-2**										
**Gender**	Male	*Reference category*								
	Female	3.19 (1.22–8.34)*	3.45 (1.12–10.67)*	1.23 (0.73–2.05)	1.47 (0.87–2.49)	1.27 (0.75–2.15)	0.67 (0.37–1.22)	0.75 (0.30–1.84)	1.82 (1.15–2.91)*	1.17 (0.73–1.87)
	Unknown^1^	^	^	^	^	^	^	^	^	^
**Age**	50–59	*Reference category*								
	60–69	0.48 (0.15–1.56)	0.60 (0.19–1.90)	0.89 (0.48–1.66)	0.98 (0.53–1.81)	1.18 (0.63–2.22)	1.17 (0.60–2.30)	0.75 (0.28–1.96)	1.14 (0.66–1.96)	1.12 (0.65–1.91)
	70–74	0.22 (0.07–0.66)**	0.53 (0.16–1.75)	0.43 (0.22–0.81)**	0.79 (0.41–1.52)	1.22 (0.61–2.44)	0.87 (0.40–1.89)	0.44 (0.13–1.49)	1.07 (0.59–1.96)	0.65 (0.35–1.22)
	Unknown^1^	^	^	0.41 (0.06–2.62)	0.40 (0.06–2.61)	0.85 (0.09–8.05)	3.28 (0.50–21.50)	^	0.93 (0.15–5.95)	5.83 (0.62–54.5)
**SES**	ABC1	*Reference category*								
	C2DE	0.37 (0.16–0.86)*	0.23 (0.08–0.65)**	0.73 (0.44–1.20)	0.51 (0.30–0.86)**	0.77 (0.45–1.31)	1.25 (0.70–2.22)	2.05 (0.83–5.09)	0.77 (0.48–1.22)	0.87 (0.55–1.39)
	Unknown^1^	0.11 (0.01–1.25)	^	1.19 (0.12–11.49)	0.34 (0.05–2.21)	1.97 (0.31–12.63)	3.81 (0.58–25.15)	4.27 (0.39–46.11)	0.28 (0.03–2.68)	0.37 (0.04–3.48)
**Ethnicity** ^2^										

OR = odds-ratio; CI = confidence intervals; SES = socioeconomic status (social grade).

*p ≤ 0.05.

**p ≤ 0.01.

^†^Binary outcome variables (aware/not aware) for the decoy items were derived as per the other health conditions, except that “*not more likely*” responses were coded aware and “*more likely*” and “*don’t know*” responses were coded not aware, because for the decoy items “*not more likely*” is the correct response.

^1^For example, respondent replied “don’t know” or declined to answer.

^2^Due to its small size, the non-white group was showing collinearity with the outcome and was therefore automatically excluded from the analysis by the statistical software (Stata 15.0).

^Perfectly predicted outcome and was therefore automatically excluded from the model by the statistical software (Stata 15.0).

#### Phase-2: Exclusively-T2DM sample

##### Participants

*N* = 319 people with T2DM (9.4% of 3,387 50–74 year olds who answered the survey question about diabetes status); sample characteristics are summarized in Table 1.

##### Awareness that T2DM is a risk factor for breast and colorectal cancer

To the open survey question, just 1.6% of respondents recalled that people with T2DM have an increased risk of cancer and, as in phase-1, no respondent specified *breast* or *bowel* cancer. In contrast, 33.9% and 34.2% of respondents recalled heart and eye problems (including sight loss), respectively, which were the two most frequent replies to the open question. Responses to the closed questions were comparable to those from the T2DM-subgroup in phase-1; as Table 2 shows, awareness was lowest for breast (7.2%) and bowel (19.4%) cancer, and also low for dementia (23.8%), though high for heart disease and nerve damage (both >70%), and very high for sight loss and foot problems (both >90%). As in phase-1, including among the T2DM-subgroup, breast and bowel cancer were the health conditions the greatest number of respondents incorrectly said were *not more likely* in people with T2DM. The pattern of responses to the decoy items were comparable to those obtained in phase-1. As in phase-1, including among the T2DM-subgroup, more respondents wrongly believed T2DM increases prostate cancer risk (12.9%) than correctly knew T2DM does confer increased risk of breast cancer (7.2%).

##### Sociodemographic correlates of awareness

As Table 3 shows, female gender, younger age (50–59 vs. 70–74), and higher SES were significantly associated with greater awareness of some of the diabetes-related health conditions, though no sociodemographic variables were significantly associated with awareness of T2DM-increased risk of breast or bowel cancer. Female gender was significantly associated with correct answers to the cervical cancer decoy item.

### Study 2: Diabetes Websites Review

#### Sample of websites

As Table 4 shows, 21 diabetes websites and 4 health-related websites with a diabetes section, were identified in the internet searches and judged eligible for review. The sample includes the websites of a range of regional, national, and international organizations, including care providers and charities, located in Britain and other higher-income countries including America and Australia.

**Table 4:** Rate of Inclusion of Cancer, Relative to Other Conditions, In Diabetes Website Sections About Associated Health Conditions in Study 2

		Listed in an evident site section about diabetes-related health conditions					
Website name	Homepage URL†	Sight loss	Foot problems	Heart disease	Nerve damage	Dementia	Cancer
1. American Diabetes Association	diabetes.org	Y	Y	Y	Y	N	N
2. Diabetes Australia	diabetesaustralia.com.au	Y	Y	Y	N	N	N
3. DiabetesCare.net	diabetescare.net	Y	Y	Y	Y	N	N
4. Diabetes.co.uk	diabetes.co.uk	Y	Y	Y	Y	Y	Y Breast and Bowel*
5. Diabetes Education Online	dtc.ucsf.edu	Y	Y	Y	Y	N	N
6. Diabetes NSW & ACT	diabetesnsw.com.au	Y	Y	Y	Y	N	N
7. Diabetes Self Caring	diabetesselfcaring.com	Y	Y	Y	Y	Y	N
8. Diabetes Self-Management	diabetesselfmanagement.com	Y	Y	Y	Y	N	Y Bowel**
9. Diabetes UK	diabetes.org.uk	Y	Y	Y	Y	N	Y Breast and Bowel*
10. diaTribe Learn	diatribe.org	Y	Y	Y	Y	N	N
11. Healthline [NDS]	healthline.com/health/diabetes	Y	Y	Y	Y	Y	N
12. International Diabetes Federation	idf.org	Y	Y	Y	Y	N	N
13. Johns Hopkins Patient Guide to Diabetes	hopkinsdiabetesinfo.org	Y	Y	Y	Y	N	N
14. Know Diabetes	knowdiabetes.org.uk	Y	Y	Y	N	N	N
15. Medical News Today [NDS]	medicalnewstoday.com/articles/323627	Y	Y	Y	Y	N	N
16. My Diabetes My Way	mydiabetesmyway.scot.nhs.uk	Y	Y	Y	N	Y	N
17. National Health Service (UK) [NDS]	nhs.uk/conditions/diabetes/	Y	Y	Y	Y	N	N
18. National Institute of Diabetes and Digestive and Kidney Diseases	niddk.nih.gov	Y	Y	Y	Y	Y	Y**
19. British Heart Foundation [NDS]	bhf.org.uk/informationsupport/risk-factors/diabetes	Y	N	Y	Y	Y	N
20. Diabetes Education Scotland^1^	diabeteseducationscotland.org.uk	No section					
21. Diabetes Research and Wellness Foundation	drwf.org.uk	No section					
22. Edinburgh Centre for Endocrinology and Diabetes	edinburghdiabetes.com	No section					
23. FreeStyle (Abbott’s Diabetes Care division)	freestylediabetes.co.uk	No section					
24. Leicester Diabetes Centre	leicesterdiabetescentre.org.uk	No section					
25. Swindon Diabetes (National Health Service)	swindondiabetes.co.uk	No section					

NDS = not diabetes-specific website.

†Archived URLs for the webpages reviewed for this stage-one of the website analyses are available in Electronic Supplementary Material 1.

^1^Website no longer accessible.

*List cancer-protective behaviours.

**Listed cancer-protective behaviours include cancer screening.

#### Inclusion of cancer in an evident site section about diabetes-related health conditions

Of the 25 websites, 19 (19/25 = 76%) were judged to have an evident section about diabetes-related health conditions, titled, for example, *diabetes complications* and *body parts affected by diabetes*. Four of these sections about diabetes-related health conditions included cancer, with three specifying *bowel* cancer (3/19 = 16%) and two *breast* cancer (2/19 = 11%). In comparison, site sections about diabetes-related health conditions all or nearly all included sight loss, heart disease (both 19/19 = 100%) and foot problems (18/19 = 95%), and most included nerve damage (16/19 = 84%), though relatively few included dementia (6/19 = 31%). All four websites that included cancer also noted, within the same section, cancer-protective behaviors (e.g., healthy diet, physical activity, and not smoking), and on two of the four sites these included cancer screening.

#### Cancer information contained elsewhere on the site

Though just four websites included cancer in their section on diabetes-related health conditions, all four sites contained more information, and an additional eight of the 21 diabetes-specific websites (websites#1, 2, 3, 6, 7, 10, 12, 24; see Table 4) contained some information about breast and/or CRC risk elsewhere on the site. Similarly, both sites that listed cancer screening among cancer-protective behaviors contained more information about this elsewhere on the site, and a further eight of the 21 diabetes-specific websites (websites#1, 2, 3, 4, 6, 9, 10, 13; see Table 4) contained some reference to cancer screening somewhere on the site. In the main, cancer risk and cancer screening information elsewhere on the sites was located on more peripheral pages, such as articles in “*news*” sections reporting on diabetes research and professional conferences (e.g., website#10), blog posts (e.g., website#6), or pdfs of journal papers aimed at and in some cases labelled as *“for professionals*” (e.g., website#18).

## Discussion

### Key Findings

This paper reports the first investigations, in Britain and internationally, of public awareness that T2DM increases the risk of developing breast and CRC, and of the rate of provision of this information from diabetes care providers and organizations on their websites. There were three key findings: (1) there was low public awareness that T2DM is a risk factor for breast and CRC, compared to much higher awareness of other diabetes-related health conditions; (2) awareness of T2DM-increased cancer risk was comparably low among people with and without T2DM, in contrast to other diabetes-related health conditions for which awareness was significantly higher among people with T2DM than those without; and contributory to understanding these findings, (3) few of the sampled diabetes websites included breast and CRC in an evident site section about diabetes-related health conditions, and fewer still included cancer screening among any noted cancer-protective behaviors.

The first study found low awareness of T2DM-increased CRC risk, which is consistent with previous research showing diabetes is the least well-recognized CRC risk factor assessed by the Bowel-CAM [31–33]. Our study shows that low awareness of CRC risk stands in contrast to much higher public awareness of other diabetes-related health conditions (e.g., sight loss, foot problems), save for dementia, for which awareness was also low, consistent with previous research examining dementia risk factor knowledge [55, 56]. Extending prior research, we found there is also very low public awareness of T2DM-increased breast cancer risk, including among women, despite breast cancer being the most common female malignancy in Britain [57]. This finding is consistent with previous research showing public under-appreciation of lifestyle-related versus heritable factors in the etiology of breast cancer compared to other malignancies such as lung and skin cancers [30, 58, 59]. Awareness of T2DM-related cancer risk was lower for breast than CRC, though the magnitude of increased risk is estimated to be lower for breast than CRC [6–8]. Nevertheless, fewer survey respondents correctly knew about T2DM-increased breast cancer risk than wrongly believed T2DM increases prostate cancer risk, despite an *inverse* association between T2DM and decoy item prostate cancer [6, 7]. It is notable that while most respondents said they *don’t know* if T2DM increases the risk of breast and bowel cancer, a sizeable proportion thought that they did know yet incorrectly believed these cancers are *not more likely* among people with T2DM.

Importantly, study 1 also shows there is low awareness of T2DM-increased cancer risk among people with and without T2DM. In phase-1 only 12% and 26% of respondents with T2DM were aware of T2DM-increased risk of breast cancer and CRC respectively, and similarly low figures were replicated in phase-2 with an exclusively-T2DM sample (7% and 19% of respondents respectively). Awareness was significantly higher among people with T2DM than those without for all diabetes-related health conditions examined in phase-1, except breast cancer and CRC. Controlling for diabetes status, in both phases of study 1, one or more of female gender, younger age and higher SES were significantly associated with greater awareness of several of the diabetes-related health conditions and correct answers to the decoy items, which is consistent with previous findings concerning sociodemographic correlates of health-related knowledge [29, 31–33, 47, 55]; an exception is that female respondents in phase-1 were less likely to know about breast and bowel cancer risk. Future research might examine clinical correlates (e.g., familial and personal history of cancer, obesity) of awareness of T2DM-increased cancer risk. To our knowledge, this is the first study to examine awareness of T2DM-increased cancer risk among people with T2DM. Future research should seek to replicate our survey findings and explore generalizability to other countries.

The second study found that few of the reviewed websites with an evident section about diabetes-related health conditions included breast or CRC there, and fewer still included cancer screening among any noted cancer-protective behaviors. Furthermore, though content analysis was outside the scope of this study, we note that the cancer information provided was limited. For example, with regard to cancer screening, one site simply noted “*getting recommended cancer screenings can help prevent cancer*” (website#18). Sites that included cancer in their diabetes-related health problems section, and interestingly several that did not, contained cancer risk and/or screening information in other more peripheral site areas like news and blog sections; website users are arguably unlikely to see this content unless regular readers of news and blog items, or they purposively site-search for cancer-related information, indicating they already have awareness of T2DM-increased cancer risk or have cancer. In contrast to cancer, sight loss, foot problems, heart disease, and nerve damage were included in all or nearly all site sections about diabetes-related health conditions; dementia, like cancer, was included on relatively few sites.

The findings of the second study provide a potential contributing explanation for the findings of the first study. Study 2 shows that, despite calls for a more holistic view of diabetes complications [8, 35], diabetes care providers and organizations still largely centre their key information provision on classical vascular complications. Thus, low public awareness of T2DM-increased breast and CRC risk may be partly due to limited information provision regarding T2DM-increased cancer risk from diabetes care providers and organizations. Indeed, the health conditions less frequently included in the diabetes-related health problems section of the reviewed websites are the same as those for which there was low public awareness, and vice versa. Importantly, the findings of study 2 also provide a potential avenue for addressing the low knowledge of T2DM-increased cancer risk seen in study 1. An effective way to raise public and patient awareness of T2DM-increased cancer risk may be for diabetes education about T2DM-related health risks to include cancer; we consider this further below. Future research should examine why cancer risk and screening do not yet feature more prominently in diabetes-related health information. It may be, for example, that care providers have low awareness of T2DM-increased breast and CRC risk; or low acceptance of T2DM as a *significant independent* cancer risk factor; or are concerned they may overwhelm patients by promoting cancer screening on top of multiple other diabetes self-care activities, especially as conceptually and empirically high experienced treatment burden is associated with lower treatment adherence [60–62].

### Strengths and Limitations

Suls and colleagues recently urged for behavioral medicine to move away from the dominant, siloed “one-condition-at-a-time” approach, and for health psychology to contribute to prevention and care of the growing challenge of multimorbidity [63]. In line with this call, a strength of this research is its dual-condition focus, in seeking to understand awareness and provision of screening relevant information about one health condition (cancer) in the context of another, predisposing chronic illness (T2DM). This research also has several methodological strengths, including: (1) use of both open (recall) and closed (recognition) questions to examine awareness; consistent with previous research [e.g., 29, 47] respondents showed better recognition than recall, as the latter places greater demands on memory and motivation; (2) inclusive survey methodology (e.g., random digit dialing, telephone interviewing); (3) recruitment of a large British-representative sample with a proportionate T2DM-subgroup and, to replicate findings among this subgroup, recruitment of a further exclusively-T2DM sample; (4) little missing data (i.e., decline to answer); and (5) review of 25 top-ranking websites, most likely to be accessed by the UK public and including those of leading diabetes care providers and charities, with 100% of the two-stage review process undertaken by two or more researchers independently.

This research inevitably has some limitations. Notably, T2DM status was self-reported, though research shows substantial agreement between self-report and medical-record data for diabetes [64, 65], and in both phases of study 1 the proportion of people with T2DM (8.6% and 9.4% respectively, of survey respondents asked about T2DM-status) corresponds with available British T2DM prevalence by age figures [2, 66]. We acknowledge that the survey methodology, though highly inclusive in not requiring written or digital literacy, still excluded some minority groups (e.g., British-resident non-English speakers; people without a telephone). Study 2 was restricted to examining online information about diabetes-related health conditions, though there is no reason to think that diabetes care providers and organizations with additional dissemination channels (e.g., print-leaflets, helplines) would offer materially different information via these channels to that which is on their website.

### Implications and Clinical Recommendations

Our findings show a need to raise public awareness of T2DM-increased breast and CRC risk (study 1) and indicate one potential way to help do this (study 2). For people with T2DM, greater awareness of their increased cancer risk is necessary for informed decision-making about cancer screening and may positively influence cancer screening intentions and uptake [23–27], plus benefit motivations for other health behaviors which promote T2DM remission. Though awareness-raising is priority among people with T2DM, there may be benefits to increasing awareness among the public generally, as this includes people with prediabetes, and family members of people with and at risk of T2DM, and research shows an influential role of family on health behaviors [67–70].

Broadening education about diabetes-related health conditions to include cancer is a potential avenue to raising awareness of T2DM-increased cancer risk. Although vascular complications are rightly the principal focus of diabetes education on associated health risks, we share the opinion of those who urge that cancer also warrants some attention [12, 35–37], in high-income countries at least. In affluent countries, cancer is a high-incidence disease (e.g., in the UK one in two people develop cancer in their lifetime [71]), with breast and CRC among the most common malignancies [57], and for people with T2DM the risk is elevated [6–8]. Furthermore, decreasing rates of vascular complications mean that in some high-income countries cancer is now the most common cause of death among people with diabetes and the leading contributor to the gap in death rates between people with and without diabetes [3, 12, 13]. Importantly, breast and CRC screening can enable earlier-stage cancer detection which has significant survival benefits [14–17] yet, in countries with national cancer screening programs, routinely offered cancer screening opportunities are underused by people with T2DM [18–21]. It is noteworthy that dementia, another so-termed “emerging complication” of T2DM, featured on slightly more of the reviewed website sections on diabetes-related health conditions than cancer did, despite there being no dementia screening opportunities akin to those for breast and CRC.

Bringing information about T2DM-increased cancer risk, and cancer screening promotion, under the wing of diabetes care could be an effective strategy for raising awareness and increasing informed uptake of cancer screening among people with T2DM. Diabetes self-care education efforts are having some degree of success, at least in the UK, given the high awareness of health problems like sight loss and foot problems shown in study 1; high uptake of retinopathy screening, which exceeds that for cancer screening [72]; and large declines in vascular disease death rates among people with diabetes [3, 12]. As a minimum, more diabetes care providers and organizations should include cancer risk and cancer screening information, and more centrally and comprehensively, in their information about diabetes-related health conditions. Diabetes clinicians and primary care doctors could include breast and CRC in patient conversations about T2DM-related health problems and highlight cancer screening as an important part of diabetes self-care; past research attests to the positive influence of clinician recommendation upon cancer screening intentions and uptake [73]. Such measures have the potential for wide reach, are rapidly implementable and, crucial in COVID-impacted economies, are affordably low-cost. In future research, we plan to examine the feasibility and impact of incorporating into diabetes self-care education various strategies to promote informed decision-making for, and uptake of, breast and CRC screening among people with T2DM.

## Conclusions

Study 1 shows that there is low public awareness that T2DM increases the risk of developing breast and CRC, including among people with T2DM. This contrasts with other diabetes-related health conditions, of which there is much higher awareness, and significantly higher awareness among people with T2DM than those without. Study 2 shows that few diabetes care providers and organizations currently include information on cancer risk, and fewer still cancer screening, in their key online information section about diabetes-related health conditions. The findings of study 2 provide a potential contributing explanation for, and avenue for addressing, the findings of study 1. Low public and patient awareness that T2DM increases cancer risk may be partly due to limited information provision regarding T2DM-increased cancer risk from diabetes care providers and organizations. A promising strategy for raising awareness of T2DM-increased cancer risk and uptake of cancer screening among people with T2DM, may be to include provision of cancer risk information and promotion of cancer screening under the wing of diabetes care, which is consistent with more integrated, holistic health care amid the growing challenge of multimorbidity.

## Supplementary Material

kaac068_suppl_Supplementary_Material
