# Supplementary material for: Increased Breast and Colorectal Cancer Risk in Type 2 Diabetes: Awareness Among Adults With and Without Diabetes and Information Provision on Diabetes Websites
Source: Ann Behav Med. 2023 Mar 9;57(5):386–98. doi: 10.1093/abm/kaac068 (PMC10122099; doi:10.1093/abm/kaac068)
Supplement: kaac068_suppl_Supplementary_Material [file kaac068_suppl_supplementary_material.docx]

**Electronic Supplementary Material 1**

Archived URLs for the webpages reviewed for stage-one of the website analysis (see Table 4 in the paper).

| **Website name** | **Archived URL(s)** |
| --- | --- |
| 1. American Diabetes Association | <https://web.archive.org/web/20220613160559/https://www.diabetes.org/diabetes> |
| 2. Diabetes Australia | <https://web.archive.org/web/20220613161128/https://www.diabetesaustralia.com.au/living-with-diabetes/preventing-complications/> |
| 3. DiabetesCare.net | <https://web.archive.org/web/20220613162117/http://www.diabetescare.net/management/complications> |
| 4. Diabetes.co.uk | - <https://web.archive.org/web/20220613163248/https://www.diabetes.co.uk/diabetes-complications/diabetes-complications.html> - <https://web.archive.org/web/20220620111949/https://www.diabetes.co.uk/how-to/avoid-diabetes-complications.html> - <https://web.archive.org/web/20220620111923/https://www.diabetes.co.uk/diabetes-complications/screening-for-diabetic-complications.html> - <https://web.archive.org/web/20220620112041/https://www.diabetes.co.uk/diabetes-complications/heart-disease.html> |
| 5. Diabetes Education Online | <https://web.archive.org/web/20220613163658/https://dtc.ucsf.edu/living-with-diabetes/complications/individual-complications/> |
| 6. Diabetes NSW & ACT | <https://web.archive.org/web/20220613164127/https://diabetesnsw.com.au/living-with-diabetes/complications-of-diabetes/> |
| 7. Diabetes Self Caring | - <https://web.archive.org/web/20220613171606/https://www.diabetesselfcaring.com/body-parts-affected-by-diabetes/> - <https://web.archive.org/web/20220613171606/https://www.diabetesselfcaring.com/body-parts-affected-by-diabetes/page/2/> - <https://web.archive.org/web/20220613171606/https://www.diabetesselfcaring.com/body-parts-affected-by-diabetes/page/3/> |
| 8. Diabetes Self-Management | - <https://web.archive.org/web/20220620113613/https://www.diabetesselfmanagement.com/education/diabetes-complications/page/2/> - <https://web.archive.org/web/20220620113702/https://www.diabetesselfmanagement.com/education/diabetes-complications/page/3/> - <https://web.archive.org/web/20220620113935/https://www.diabetesselfmanagement.com/education/diabetes-complications/page/5/> - <https://web.archive.org/web/20220620114024/https://www.diabetesselfmanagement.com/education/diabetes-complications/page/6/> - <https://web.archive.org/web/20220620114127/https://www.diabetesselfmanagement.com/education/diabetes-complications/page/8/> - <https://web.archive.org/web/20220620115004/https://www.diabetesselfmanagement.com/education/diabetes-complications/page/32/> - <https://web.archive.org/web/20220620115037/https://www.diabetesselfmanagement.com/education/diabetes-complications/page/33/> |
| 9. Diabetes UK | - <https://web.archive.org/web/20220613174334/https://www.diabetes.org.uk/guide-to-diabetes/complications> - <https://web.archive.org/web/20220620115416/https://www.diabetes.org.uk/diabetes-the-basics/related-conditions> - <https://web.archive.org/web/20220620115643/https://www.diabetes.org.uk/diabetes-the-basics/related-conditions/diabetes-and-cancer> |
| 10. diaTribe Learn | <https://web.archive.org/web/20220613174718/https://diatribe.org/diabetes-complications> |
| 11. Healthline [NDS] | <https://web.archive.org/web/20220613174925/https://www.healthline.com/health/diabetes> |
| 12. International Diabetes Federation | <https://web.archive.org/web/20220620121241/https://idf.org/aboutdiabetes/complications.html> |
| 13. John Hopkins Patient Guide to Diabetes | <https://web.archive.org/web/20220613180234/https://hopkinsdiabetesinfo.org/complications/> |
| 14. Know Diabetes | <https://web.archive.org/web/20220613180441/https://www.knowdiabetes.org.uk/know-more/feet-heart-eyes-and-kidneys/> |
| 15. Medical News Today [NDS] | <https://web.archive.org/web/20220613180658/https://www.medicalnewstoday.com/articles/323627> |
| 16. My Diabetes My Way | - <https://web.archive.org/web/20220613181055/https://mydiabetesmyway.scot.nhs.uk/know-more/my-complications/> - <https://web.archive.org/web/20220621081148/https://mydiabetesmyway.scot.nhs.uk/know-more/my-complications/heart-and-vascular-disease/> - <https://web.archive.org/web/20220620122116/https://mydiabetesmyway.scot.nhs.uk/resources/files/living-with-diabetes-and-dementia/> |
| 17. National Health Service (UK) [NDS] | <https://web.archive.org/web/20220613181420/https://www.nhs.uk/conditions/type-1-diabetes/living-with-type-1-diabetes/avoiding-complications/> |
| 18. National Institute of Diabetes and Digestive and Kidney Diseases | <https://web.archive.org/web/20220613182017/https://www.niddk.nih.gov/health-information/diabetes/overview/preventing-problems> |
| 19. British Heart Foundation [NDS] | <https://web.archive.org/web/20220614084554/https://www.bhf.org.uk/informationsupport/risk-factors/diabetes#Heading9> |
| 20. Diabetes Education Scotland | Website became no longer accessible prior to creating archived URLs in June 2022 |
| 21. Diabetes Research and Wellness Foundation | No evident site section about diabetes-related health conditions  <https://web.archive.org/web/20220614085551/https://www.drwf.org.uk/understanding-diabetes/> |
| 22. Edinburgh Centre for Endocrinology and Diabetes | No evident site section about diabetes-related health conditions  <https://web.archive.org/web/20220614085412/https://www.edinburghdiabetes.com/information-faqs> |
| 23. FreeStyle (Abbott’s Diabetes Care division) | No evident site section about diabetes-related health conditions  <https://web.archive.org/web/20220614085918/https://freestylediabetes.co.uk/> |
| 24. Leicester Diabetes Centre | No evident site section about diabetes-related health conditions  <https://web.archive.org/web/20220614091132/https://www.leicesterdiabetescentre.org.uk/mydiabetes> |
| 25. Swindon Diabetes (National Health Service) | No evident site section about diabetes-related health conditions  <https://web.archive.org/web/20220614091504/https://www.swindondiabetes.co.uk/support/> |

NDS = not diabetes-specific website
